# Novel heterozygous mutation in the SHOX gene leading to familial idiopathic short stature: A case report and literature review

**DOI:** 10.1097/MD.0000000000035471

**Published:** 2023-10-13

**Authors:** Lifang Liu, Junsheng Li, Jiarui Li, Hui Hu, Jiao Liu, Ping Tang

**Affiliations:** a Jiaxing Maternity and Children Health Care Hospital/The Affiliated Women and Children’s Hospital of Jiaxing University, Jiaxing, Zhejiang, China; b Lishui Maternal and Child Health Hospital, Lishui, Zhejiang, China; c Department of Orthopaedic Surgery, The First Affiliated Hospital, Zhejiang University School of Medicine, Hangzhou, China.

**Keywords:** genetic etiology, idiopathic short stature, SHOX gene, whole-exome sequencing

## Abstract

**Background:**

The pathogenic mutation of short stature homeobox (SHOX) gene is one of the main genetic causes of short stature in children, with an incidence rate of 1/1000~1/2000 and the main clinical manifestations are short stature and (or) limb skeletal abnormalities. SHOX gene mutations are mostly large deletions of regulatory sequence genes, while exon mutations are relatively rare. The pathogenic rate of mutations occurring in exon 5 is only 1/50 000~1/100 000. This study reviewed the clinical data of a child with SHOX gene mutation in exon 5, and analyzed the clinical phenotype, pathogenesis, diagnosis, treatment and prognosis of SHOX gene mutation in combination with relevant literature at home and abroad.

**Case presentation:**

The patient was an 8-year-old girl with a height of 105.2 cm (−4.31 standard deviations). Her sitting height/height ratio was 56.8% (>55.5%), and she exhibited high-arched palate, irregular dentition, micrognathia, short fingers, and a normal growth hormone stimulation test. Whole-exome sequencing was performed, and Sanger sequencing was used for site validation. The sequencing results revealed a heterozygous mutation of c.577G > A in exon 5 of the SHOX gene, inherited from the father. The clinical symptoms of the proband were consistent with the phenotype of short stature idiopathic familial associated with SHOX gene mutations. The father, grandfather, uncle, and sister of the proband all had the c.577G > A heterozygous mutation. Therefore, the clinical diagnosis was childhood short stature caused by SHOX gene defects. The SHOX: c.577G > A mutation is likely to be the genetic etiology of familial idiopathic short stature in this family, and this novel mutation enriches the mutation spectrum of the SHOX gene.

**Conclusion:**

This is the first case report of familial idiopathic dwarfism caused by mutation at the c.577G > A locus of exon 5 of SHOX gene in the world. This novel mutation enriches the mutation spectrum of the SHOX gene. It is important to emphasize genetic testing, including the SHOX gene, in patients with familial idiopathic short stature and to provide timely growth hormone therapy to individuals with short stature caused by SHOX gene mutations in order to improve their adult height.

## 1. Introduction

Short stature refers to individuals whose height is below 2 standard deviations (−2 SD) of the average height for their gender, age, and ethnicity, or below the 3rd percentile.^[1]^ Short stature idiopathic familial (ISS) accounts for approximately 70% of cases. Linear growth in the human body is a programmed developmental process influenced by both genetic and environmental factors. Currently, hundreds of genes have been identified to be associated with height, among which genes such as short stature homeobox (SHOX), SOX9, COL2A1, and FGFR3 significantly affect height.^[2,3]^

The SHOX gene, short for short (SHOX, MIM 312865), is one of the major genetic causes of short stature in children, with an incidence of 1/1000 to 1/2000.^[4]^ Clinically, it is characterized by short stature and/or skeletal abnormalities in the limbs (mesomelic dysplasia and Madelung deformity).^[5]^ Based on different clinical phenotypes, it is classified into Langer mesomelic dysplasia, Leri-Weill dyschondrosteosis, and ISS.^[6]^ Mutations in the SHOX gene often involve large deletions in regulatory sequences, while exon mutations are less common, with a pathogenicity rate of only 1/50,000 to 1/100,000 in mutations occurring in exon 5.^[7]^ In this study, we retrospectively analyzed the clinical data of a patient with short stature caused by a mutation in exon 5 of the SHOX gene and combined it with relevant domestic and international literature to analyze the clinical phenotype, pathogenesis, diagnostic and treatment methods, and prognosis of SHOX gene mutations.

## 2. Patients and Methods

### 2.1. Case presentation

The patient was an 8-year-old girl who presented to the Pediatric Growth and Development Clinic in August 2021 with a complaint of “slow growth.” With informed consent from the patient guardian, we collected her medical history and physical examination data, conducted laboratory and imaging examinations, and performed whole-exome sequencing and Sanger familial validation. This study was approved by the Medical Ethics Committee of our institution, and the research process complied with ethical requirements. Informed consent was obtained from the patient for publication of this case report details.

### 2.2. Physical examination

The patient height, weight, growth rate, body mass index, sitting height/height ratio, finger length, and secondary sexual characteristics (Tanner staging) were measured and recorded.

### 2.3. Laboratory and imaging examinations

Liver and kidney function, electrolytes, glucose and lipid metabolism, thyroid function, adrenocorticotropic hormone, cortisol, sex hormones, tumor markers were tested. A growth hormone stimulation test was performed using arginine and levodopa. Additionally, ultrasound (thyroid, parathyroid, liver, gallbladder, pancreas, spleen, kidneys, adrenal glands, uterus, and adnexa), bone age assessment, pituitary MRI, and full-length lateral spine X-ray were conducted. Bone age assessment was performed using the Greulich-Pyle method.

### 2.4. DNA sequencing

Sequencing was performed using the Illumina NovaSeq platform. SureSelect XT Human All Exon V6 (Agilent) was used for target region capture and library construction. The samples were sequenced using paired-end (2 × 150 bp) sequencing. Sanger sequencing was used for familial validation of the identified variants. The primer sequences for PCR were as follows: F: CATCTCTCTCTGCTTCTCCCCAA; R: GAGTGTCAGGATGCGGCAGCA; F: ATAAAGGTGGGTGTCGGGAC; R:CGGCAGCAAATAGGGGAA.

### 2.5. Bioinformatics analysis

The high-throughput targeted gene sequencing data (Fastq files) were subjected to low-quality filtering. The filtered sequences were aligned to the human reference genome using the BWA sequence alignment method. GATK was used to identify mutation sites in the target sequences. The Annovar annotation software was used to annotate the mutation sites to public mutation databases. The impact of the mutations on protein function was predicted based on the frequency of the mutation in the normal population, sequence conservation, amino acid changes caused by the mutation, and the position of the mutation in the protein structure. Finally, based on the clinical phenotype of the sample and following the American society of medical genetics and genomics (ACMG) standards and guidelines, the pathogenicity of the mutations was interpreted. The reference databases used were as follows: HGMD (Human Gene Mutation Database): http://www.hgmd.cf.ac.uk/ac/index.php; ClinVar: https://www.ncbi.nlm.nih.gov/clinvar/; GnomAD (Genome Aggregation Database): https://gnomad.broadinstitute.org/; ExAC (Exome Aggregation Consortium): http://exac.broadinstitute.org; MutationTaster and PolyPhen-2 (tools for predicting the functional impact of gene mutations): http://www.mutationtaster.org/; http://genetics.bwh.harvard.edu/pph2/; SIFT and Provean (tools for predicting the functional impact of protein mutations): http://provean.jcvi.org/protein_batch_submit.php?species=human.

## 3. Results

### 3.1. Medical history

The patient has experienced delayed growth for 7 years, with a growth rate of 2 to 3 cm/year. Their diet, exercise, and sleep patterns are normal. There are no sensory impairments, abdominal pain, diarrhea, excessive urination, or excessive drinking. The patient has no history of recurrent infections, surgical trauma, or allergies. They have received routine vaccinations.

The patient father, sister, grandfather, grandmother, and uncle are all of short stature, while the mother height is normal (Table 1). The patient has normal motor, language, and intellectual development. The family members deny consanguineous marriage and a history of genetic disorders.

**Table 1:** Clinical data of family members.

Member clinical features	Grandfather I-1	Grandmother I-2	Mother II-1	Father II-2	Uncle II-3	Sister III -1	Proband III-2
Age (yr)	78	74	41	47	48	16	8
Gender (cm)	男	女	女	男	男	女	女
Height (cm)	157.2	148.5	153.2	160.0	160.3	149.1	105.2
Height (SDS)	−2.54	−2.24	−1.37	−2.08	−2.03	−2.04	−4.31
Distinctive facial features*	Yes	No	No	Yes	Yes	No	Yes
Brachydactyly	Yes	No	No	Yes	Yes	Yes	Yes
Early development of secondary sex characteristic	No	No	No	No	No	No	No
Madelung deformity	No	No	No	No	No	No	No

SDS = standard deviation scores.

* Distinctive facial features: misaligned teeth, small chin, high-arched palate.

### 3.2. Physical examination results

**Figure 1.**
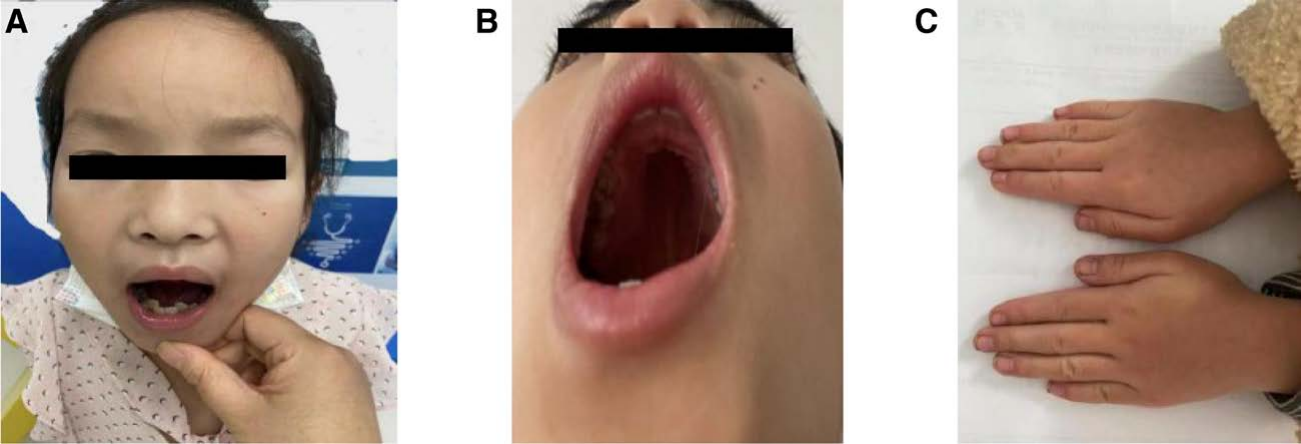
Distinctive facial features and skeletal abnormalities in the proband. (A) Misaligned teeth, small chin. (B) High-arched palate. (C) Short fingers (toes).

The patient height is 105.2 cm (−4.31 SD), weight is 15.0 kg (<−2.0 SD), body mass index is 13.5 kg/m^2^ (−1.1 SD), sitting height/height ratio is 56.8% (>55.5%), abdominal fat is 0.7 cm, teeth are misaligned, and they have a small chin (Fig. 1A); high-arched palate (Fig. 1B); short fingers (toes) (Fig. 1C); normal skin; no abnormal findings in the heart and lungs; liver and spleen not palpable below the ribs; no abnormal muscle strength or muscle tone in the limbs; normal external genitalia; Tanner stage I.

### 3.3. Laboratory and imaging examination results

Insulin-like growth factor-1 (IGF-1) is 80 µg/L (−1.8 SD), and the growth hormone stimulation test indicates a peak growth hormone level of 28.15 µg/L. Liver and kidney function, electrolyte levels, glucose and lipid metabolism, thyroid function, adrenocorticotropic hormone, cortisol, tumor markers, and full set of sex hormones are all within normal range. Peripheral blood karyotyping analysis does not show any significant abnormalities. B-ultrasound examination (thyroid, parathyroid, liver, gallbladder, pancreas, spleen, kidneys, adrenal glands, uterine appendages) does not reveal any significant abnormalities. Pituitary MRI and full-length lateral spine X-ray examination do not show any significant abnormalities. Limb X-ray examination shows normal findings without Madelung deformity. Bone age assessment indicates a bone age of 6 years for the patient.

### 3.4. Whole exon sequencing

**Figure 2.**
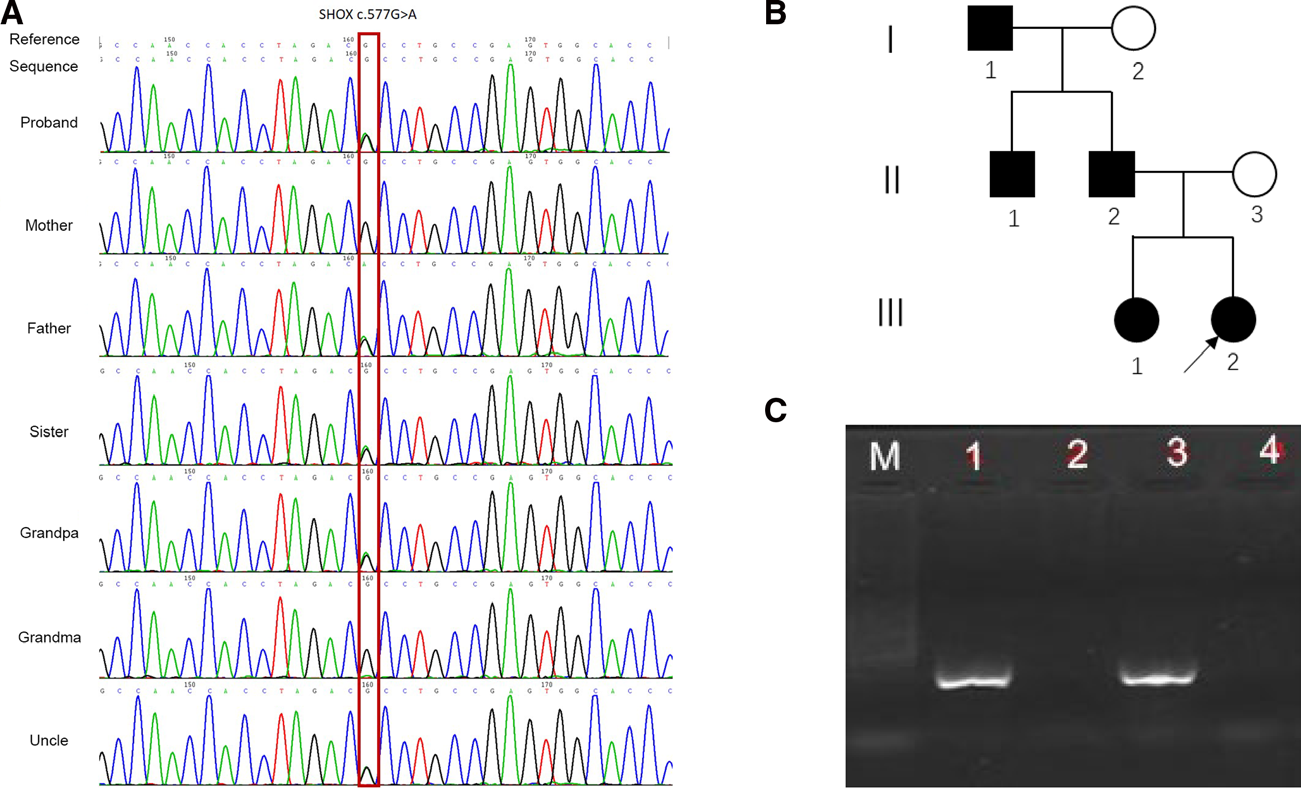
Novel heterozygous mutation of short stature homeobox (SHOX) gene. (A) Sanger sequencing results of SHOX gene in subject and family members. (B) Family tree (I-1: Grandfather of the proband; I-2: Grandmother of the proband; II-1: Uncle of the proband; II-2: The father of the proband; II-3: mother of the proband; III-1: Sister of the proband; III-2: proband [arrow]) (C) PCR electrophoresis of Sex-determining region Y protein (M: Marker; 1:grandpa; 2:grandma; 3: Positive control; 4: Negative control).

High throughput sequencing revealed the presence of NM in the proband_ 000451.3 (SHOX): c.577G > A (p.Ala193Thr) heterozygous variation, which is located in exon 5. The family verification was carried out for the SHOX: c.577G > A found by sequencing of the whole Exon group. As shown in Figure 2A, The mutation locus of the proband was consistent with that of the whole Exon group, and was inherited from the father. The father, uncle, grandfather, and sister of the proband all carry a c.577G > A heterozygous mutation, and no other tested family members have this mutation (Fig. 2B).

### 3.5. Bioinformatics analysis results

SHOX gene c.577G > A (p.Ala193Thr) mutation causes alanine at 193 to change to Threonine. The HGMD database did not include this mutation; There is one record in the ClinVar database indicating that the mutation is clinically uncertain (as of August 2021); The GnomAD database and ExAC database both show that the frequency of this mutation in the overall population is <1 ‰ (PM2); The mutation conforms to the phenomenon of genotype phenotype co segregation (PP1) in the family; The genotype and phenotype of the proband are highly consistent (PP4); The Mutation taster database predicts that it is harmful, while the Provean, SIFT, and Polyphen-2 databases predict that it is benign, resulting in inconsistent predictions. According to the guidelines of the ACMG, the clinical significance of this mutation is still unclear (ACMG: PM2, PP4, PP1). In addition, the copy number variation analysis based on the detection data of the whole Exon group of the subject and the comprehensive analysis combined with the clinical phenotype did not find any known or potentially clinically significant copy number variation related to the disease phenotype of the subject.

### 3.6. Sample verification

SHOX gene is located in the Autosome like region 1 (PAR1). It is inferred from the paternal model of grandpa grandma uncle father that the father inherited the c.577G > A locus of grandpa Y chromosome; from the pedigree model of father mother sister proband, the c.577G > A locus is located on the father X chromosome. There are contradictions before and after. Further validate the sample. A: Excluding Sanger false positives: The results of the Sanger validation again are consistent with the previous validation. B: Exclusion of grandpa and grandma mixed samples: Sex-determining region Y amplification experiment (Fig. 2C) showed that sex-determining region Y was detected in grandpa samples, but not in grandma samples, because the possibility of grandpa and grandma mixed samples could be ruled out.

### 3.7. Diagnosis and differential diagnosis

Due to the identification of the SHOX gene mutation c.577G > A (p.Ala193Thr) through whole-exome sequencing in the affected child, combined with Sanger family validation results, the final diagnosis was determined to be familial idiopathic short stature caused by a mutation in the SHOX gene. The chromosomal karyotype was normal, and uterine adnexal ultrasound and sex hormone levels were normal, ruling out Turner syndrome. The child did not have malnutrition, excluding malnutrition-induced short stature. Additionally, thyroid function, adrenocorticotropic hormone, cortisol, and sex hormone levels were normal, and the peak growth hormone was normal, thus excluding growth hormone deficiency and multiple pituitary hormone deficiencies. The child liver and kidney function, glucose and lipid metabolism, and other test results were normal, ruling out chronic systemic diseases. Pituitary MRI and tumor marker tests were normal, excluding tumor infiltration and brain damage.

### 3.8. Treatment and follow-up

The child received subcutaneous injections of recombinant human growth hormone (rhGH) at a dose of 0.15 IU/kg/day for 17 months. During this period, regular follow-ups were conducted for glucose and lipid metabolism, thyroid function, spinal radiographs in the anteroposterior and lateral views, and tumor markers. The child height increased by 11.3 cm, corresponding to an increase of 1.94 standard deviations. No thyroid dysfunction, tumors, fractures, or spinal deformities occurred.

## 4. Discussion

### 4.1. SHOX gene and diseases

The SHOX gene, also known as the short stature homeobox gene (SHOX, MIM 312865), is one of the main genetic factors leading to short stature in children.^[6]^ Its clinical manifestations include short stature and/or limb skeletal abnormalities (mesomelic and Madelung deformities). The SHOX gene consists of 7 exons and spans approximately 40 kb. The regulation of the growth plate by the SHOX gene exhibits a gene-dosage effect, and patients with SHOX defects show significant phenotypic heterogeneity.^[8–10]^ Pathogenic mutations in the gene can cause Léri-Weill dyschondrosteosis as a pseudoautosomal dominant disorder and Langer mesomelic dysplasia as a pseudoautosomal recessive disorder. They can also cause ISS with an unknown genetic pattern.^[6,11]^ It has been reported that SHOX gene mutations account for 6% to 22% of the genetic causes of ISS.^[1,12,13]^ In addition to short stature, ISS caused by SHOX gene mutations may be associated with features such as high-arched palate and mild Turner-like characteristics. Forearm abnormalities, short stature, and sitting height-to-height ratio are highly correlated with SHOX-related features.^[13–15]^ The clinical phenotype of the index case in this study, including short stature, high-arched palate, and increased sitting height-to-height ratio, is highly consistent with ISS caused by SHOX gene mutations.

To date, more than 300 mutations in the SHOX gene have been reported.^[11]^ Among them, large deletions involving both coding and non-coding regulatory sequences account for approximately 80% of the mutations, while mutations in the coding and promoter sequences account for about 20%, including substitutions, small deletions, and insertions.^[16,17]^ Mutations in the coding and promoter sequences of the SHOX gene are more commonly found in exons 2, 3, and 4, while mutations in exon 5 account for only 6.4% of the mutations in the coding and promoter sequences of the SHOX gene.^[18]^ Currently, there are no literature reports on the c.577G > A mutation in exon

### 4.2. PAR1 homologous recombination (HR)

HR of DNA, 1 of the 3 major DNA metabolic pathways including DNA replication, recombination, and damage repair, is a fundamental biological event in living organisms. It plays essential roles in various aspects such as cell growth, meiosis, gametogenesis, species evolution, DNA double-strand break repair, and genome stability maintenance.

**Figure 3.**
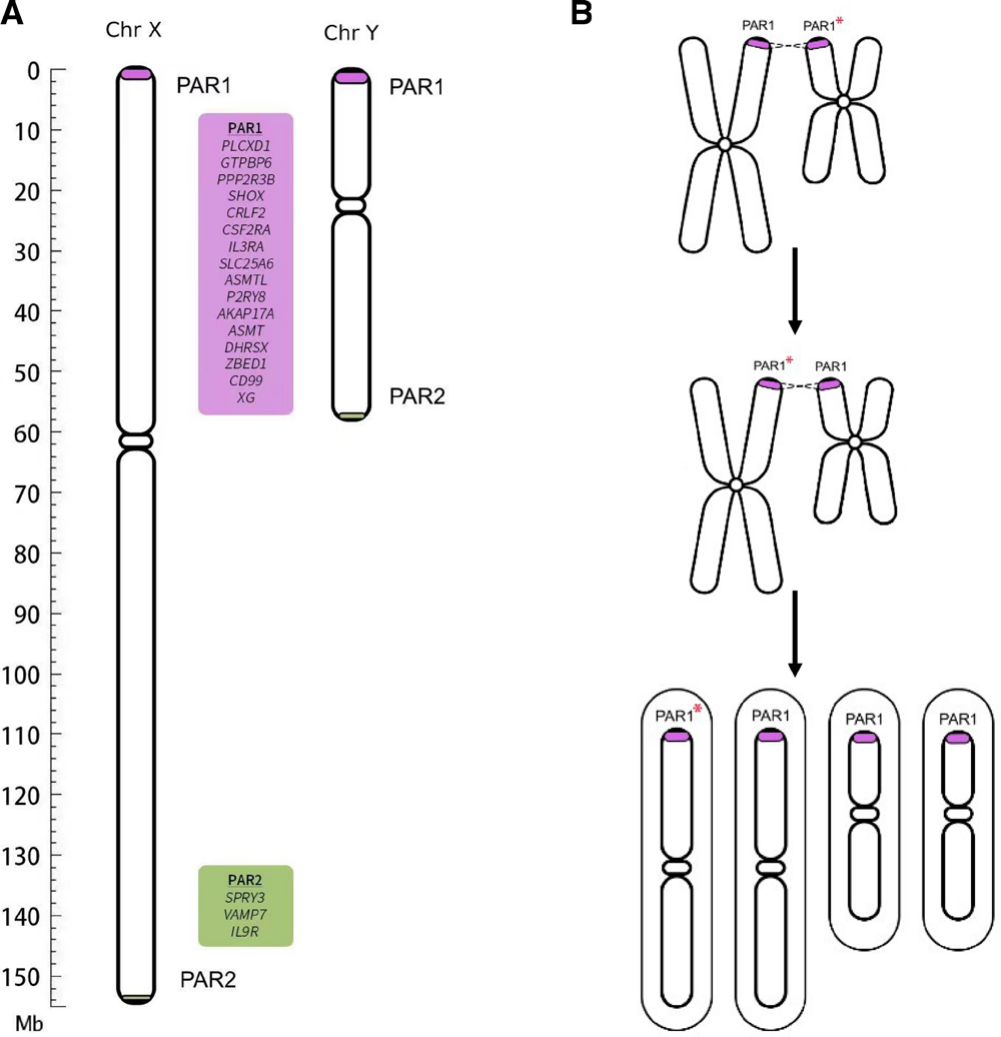
Gene distribution in the pseudo autosomal region (PAR) and recombination in the PAR1 region. (A) Distribution of known genes in PAR region of X chromosome and Y chromosome. (B) Schematic diagram of PAR1 region reorganization.

Pseudo autosomal regions (PARs) refer to certain homologous regions present on the sex chromosomes, where these regions exhibit identical or highly similar DNA sequences on the X and Y chromosomes. PAR regions are located at the ends of the X and Y chromosomes and undergo HR during meiosis, ensuring genetic balance and stability between the X and Y chromosomes. In the human genome, PAR regions are primarily divided into 2 regions: PAR1 and PAR2 (Fig. 3A). PAR1 is located at the ends of the X and Y chromosomes and spans approximately 2.6 Mb. Within this region, 24 genes have been identified, such as SHOX, IL3RA, and CSF2RA. PAR2 is situated in the middle of the X and Y chromosomes, spanning around 320 kb, and currently, 5 genes have been reported within this region, including AMELY and AMELX.^[19]^

Studies have shown that PARs undergo pairing and recombination during meiosis similar to autosomes. However, the recombination activity of PAR1 differs significantly between sexes. PAR1 is a male-specific recombination hotspot, and research on human sperm and pedigree charts indicates that the recombination rate within PAR1 aligns with a mandatory crossover in each male meiosis, rarely accompanied by a second crossover.^[20,21]^ The crossover rate within PAR1 is 17 times higher than the genome-wide average. In contrast, the female recombination rate within PAR1 is comparable to the genome-wide average.^[22,23]^ Recombination activity within PAR1 is particularly intense from the telomere end toward the region center and gradually decreases towards the pseudoautosomal boundary.^[23,24]^

Based on this, we speculate that the “contradictory” inheritance pattern observed in this pedigree may be due to recombination events occurring in the PAR1 region during the formation of the father sperm cells. This resulted in the transfer of the c.577G > A variant located on the Y chromosome to the X chromosome, which was then passed on to both daughters (Fig. 3B).

### 4.3. Treatment advances

Early studies have demonstrated the effectiveness of rhGH therapy in the treatment of ISS caused by SHOX gene mutations. In 2006, the U.S. FDA officially approved the use of rhGH for the treatment of short stature in children with SHOX gene defects not associated with growth hormone deficiency. Research has reported that rhGH treatment for ISS caused by SHOX gene mutations resulted in an average height increase of 3.5 cm in the first year and 1.9 cm in the second year. Continued treatment showed an average height improvement of > 1.3 standard deviation scores, with 41% of patients achieving heights within the normal range. The treatment efficacy was better in patients with enhancer deletions compared to those with coding gene mutations. No new adverse reactions were observed in individuals with SHOX gene defects undergoing rhGH treatment. SHOX gene mutations are often associated with accelerated bone age, early puberty, and rapid progression. For children with idiopathic short stature during puberty, in addition to rhGH treatment, gonadotropin-releasing hormone agonists can be used to suppress bone age advancement and precocious puberty.^[25]^ Furthermore, aromatase inhibitors can selectively inactivate aromatase, block aromatization reactions, inhibit estrogen production, and reduce estrogen levels in the blood, thereby slowing down bone age progression and extending the duration of rhGH treatment growth-promoting effects. The combination of rhGH and aromatase inhibitors has been shown to better improve adult height in males with advanced bone age.^[26]^

In summary, we uncovered a compound heterozygous variant in the SHOX (c.577G > A) gene in a female Chinese child with familial idiopathic short stature, which inherited from his father. These variants associated with short stature and skeletal abnormalities in the limbs, which extends the phenotype spectrum of SHOX variations.

## Acknowledgments

We are grateful to the all the patients and their families participating in this study.

## Author contributions

**Conceptualization:** Jiao Liu, Ping Tang.

**Data curation:** Junsheng Li, Jiao Liu.

**Formal analysis:** Lifang Liu, Jiarui Li.

**Funding acquisition:** Jiao Liu, Ping Tang.

**Investigation:** Lifang Liu, Junsheng Li.

**Methodology:** Junsheng Li, Jiarui Li, Hui Hu.

**Visualization:** Ping Tang.

**Writing – original draft:** Lifang Liu.

**Writing – review & editing:** Junsheng Li, Jiarui Li.
